# The Effectiveness of Mobile Phone Messaging–Based Interventions to Promote Physical Activity in Type 2 Diabetes Mellitus: Systematic Review and Meta-analysis

**DOI:** 10.2196/29663

**Published:** 2022-03-08

**Authors:** Mohammed Alsahli, Alaa Abd-Alrazaq, Mowafa Househ, Stathis Konstantinidis, Holly Blake

**Affiliations:** 1 School of Health Sciences Faculty of Medicine and Health Sciences University of Nottingham Nottingham United Kingdom; 2 Division of Health Informatics College of Health Science Saudi Electronic University Riyadh Saudi Arabia; 3 Division of Information and Computing Technology, College of Science and Engineering Hamad Bin Khalifa University Qatar Foundation Doha Qatar; 4 AI Center for Precision Health Weill Cornell Medicine-Qatar Doha Qatar; 5 Nottingham Biomedical Research Centre National Institute for Health Research Nottingham United Kingdom

**Keywords:** type 2 diabetes mellitus, physical activity, mobile phone messaging, systematic review, meta-analysis

## Abstract

**Background:**

The prevalence of type 2 diabetes mellitus (T2DM) is increasing worldwide. Physical activity (PA) is an important aspect of self-care and first line management for T2DM. SMS text messaging can be used to support self-management in people with T2DM, but the effectiveness of mobile text message–based interventions in increasing PA is still unclear.

**Objective:**

This study aims to assess the effectiveness of mobile phone messaging on PA in people with T2DM by summarizing and pooling the findings of previous literature.

**Methods:**

A systematic review was conducted to accomplish this objective. Search sources included 5 bibliographic databases (MEDLINE, Cochrane Library, CINAHL, Web of Science, and Embase), the search engine *Google Scholar* (Google Inc), and backward and forward reference list checking of the included studies and relevant reviews. A total of 2 reviewers (MA and AA) independently carried out the study selection, data extraction, risk of bias assessment, and quality of evidence evaluation. The results of the included studies were synthesized narratively and statistically, as appropriate.

**Results:**

We included 3.8% (6/151) of the retrieved studies. The results of individual studies were contradictory regarding the effectiveness of mobile text messaging on PA. However, a meta-analysis of the results of 5 studies showed no statistically significant effect (*P*=.16) of text messages on PA in comparison with no intervention. A meta-analysis of the findings of 2 studies showed a nonsignificant effect (*P*=.14) of text messages on glycemic control. Of the 541 studies, 2 (0.4%) found a nonsignificant effect of text messages on anthropometric measures (weight and BMI).

**Conclusions:**

We could not draw a definitive conclusion regarding the effectiveness of text messaging on PA, glycemic control, weight, or BMI among patients with T2MD, given the limited number of included studies and their high risk of bias. Therefore, there is a need for more high-quality primary studies.

**Trial Registration:**

PROSPERO International Prospective Register of Systematic Reviews CRD42020156465; https://www.crd.york.ac.uk/prospero/display_record.php?RecordID=156465

## Introduction

### Background

The burden of diabetes is increasing, and the number of people with type 2 diabetes mellitus (T2DM) worldwide has reached 387 million and is expected to increase to 592 million by 2035 [1]. This prevalence imposes a high and rising burden of lifelong multiorgan complications, leading to increased disability and risk of premature deaths, mainly in low- and middle-income countries [2]. A considerable amount of literature suggests that better management of T2DM delays the onset of short- and long-term complications among people diagnosed with T2DM [3-5]. Over the past decades, physical activity (PA) has been part of the first line T2DM care management [6]. PA includes all movements that increase energy use; however, there are three main types of exercise: aerobic, strength training, and flexibility work [7]. PA can help people with T2DM achieve a variety of goals, including increased vigor, improved glycemic hemoglobin control, decreased insulin resistance, increased cardiorespiratory fitness, improved lipid profile, blood pressure reduction, and maintenance of weight loss [8]. Unfortunately, patients with T2DM are less likely to engage in regular PA, with recent estimates demonstrating a lower participation rate compared with the national average [9]. There have been many attempts to explore alternative approaches to improve PA in people with T2DM, and the mobile phone messaging revolution has brought entirely new opportunities and increased access to self-management education [1]. The literature shows that text messaging–based interventions can be effective in improving health-related behaviors and bridging the gaps between patients and health care services for people living with chronic diseases [10,11]. Text messaging may be 1-way (unidirectional) or 2-way (bidirectional); they can be standardized or tailored to specific patients and sent at varied frequencies based on the intervention design [12]. Multiple meta-analyses have demonstrated the overall success of mobile phone messaging in promoting various aspects of behavior change for PA and mental health–related disorders [1,13,14].

### Research Problem and Aim

Several studies have assessed the effect of mobile text messaging on the PA of patients with T2DM. It is crucial to summarize and aggregate the findings of such studies to produce more generalizable and definitive conclusions about the effectiveness of such interventions. A total of 4 previous systematic reviews did not provide evidence from studies with text messaging interventions that specifically targeted PA. Specifically, the first review focused on the impact of education on T2DM delivered via mobile text messaging [15]. The second review assessed the effectiveness of text messaging interventions on glycated hemoglobin (HbA_1c_) in patients with T2DM, including all self-management strategies [1]. The third review identified randomized trials conducted to improve glycemic control in T2DM, which involved the delivery of behavior change content through a range of digital platforms and approaches (eg, SMS text messaging, multimedia message service, or instant messaging such as WhatsApp) [12]. The fourth review assessed the effectiveness of technology-based interventions to promote PA in T2DM; for this review, technology included mobile phones and text messages, websites, CD-ROMs, and computer learning–based technology [16]. This review was conducted approximately 7 years ago, but studies involving technology-based interventions are rapidly emerging and there may be new published evidence. Therefore, this study aims to assess the effectiveness of mobile phone messaging on PA in patients with T2DM by summarizing and pooling the findings of previous literature.

## Methods

### Overview

A systematic review was conducted and reported in accordance with the PRISMA (Preferred Reporting Items for Systematic Reviews and Meta-Analyses) statement (Multimedia Appendix 1) [17]. The protocol for this review was registered at PROSPERO (ID: CRD42020156465).

### Search Strategy

#### Search Sources

We used the following electronic databases in our search: MEDLINE, Cochrane Library, CINAHL, Web of Science, and Embase. These databases were searched on April 19, 2020, by the first author (MA). Auto alerts were set after searching the databases to conduct an automatic search weekly for 16 weeks (ending on August 9, 2020) and send us the retrieved studies. We also searched the search engine *Google Scholar* (Google Inc) to identify gray literature. To identify further studies of relevance to the review, we screened the reference lists of included studies (ie, backward reference list checking) and identified and screened studies that cited the included studies (ie, forward reference list checking).

#### Search Terms

The search terms were identified by consulting 2 experts in eHealth interventions for patients with diabetes and by checking systematic reviews of relevance to the review. These terms were chosen based on the target population (eg, type 2 diabetes, diabetes type 2, and type II diabetes), target intervention (eg, text messaging, text messages, and short messages), target outcome (eg, PA, physical exercise, HbA_1c_, and weight), and target study design (eg, trial, experiment, and randomized controlled trial [RCT]). Multimedia Appendix 2 shows the detailed search query used for searching MEDLINE.

#### Study Eligibility Criteria

The population of interest was adult patients (≥18 years) with T2DM, regardless of their gender and ethnicity. We excluded patients with type 1 diabetes mellitus, gestational diabetes, and prediabetes. The target intervention in this review was mobile phone text messages (SMS text messaging and multimedia message service), but not mobile apps, web-delivered interventions, wearables, or emails. The aim of the text messages was to improve solely PA but not diet, lifestyle, diabetic literacy, or other aspects of self-care. The primary outcomes of interest were subjectively or objectively measured PA (eg, step counts), glycemic control (eg, HbA_1c_ and fasting glucose), and anthropometric measures (eg, change in weight and BMI). Only RCTs were eligible for inclusion in this review. We considered studies published only in the English language. No restrictions were applied to the year of publication, country of publication, comparator, type of publication, or study setting.

#### Study Selection

We followed 2 steps of the study selection process. In the first step, 2 reviewers (MA and AA) independently sifted the titles and abstracts of all retrieved studies. In the second step, the 2 reviewers independently scrutinized the full texts of the studies included in the first step. In both steps, any disagreements among the reviewers were resolved through discussion and consensus. Cohen *κ* in this review indicated a very good level of interrater agreement in the first (0.88) and second step (0.95) of the selection process [18].

#### Data Extraction

Multimedia Appendix 3 shows the data extraction form that was used in this review to precisely and systematically extract the data from the included studies. A total of 2 reviewers (MA and AA) independently conducted data extraction from the included studies, and they resolved any disagreements through discussion and consensus. Cohen *κ* showed a very good level of interrater agreement among the reviewers (0.85) [18].

#### Risk of Bias Assessment

To assess the risk of bias in the included studies, we used the Risk of Bias 2 tool, which is recommended by the Cochrane Collaboration [19]. This tool assesses RCTs in terms of five domains: randomization process, deviations from intended interventions, missing outcome data, measurement of the outcome, and selection of the reported result [19]. Then, the overall risk of bias was determined for each study based on the risk of bias judgments in the five domains [19]. A total of 2 reviewers (MA and AA) independently assessed the risk of bias in the included studies, and any disagreements were resolved through discussion and consensus. Interrater agreement among the reviewers was very good (Cohen *κ*=0.86) [18]. We presented the results of the risk of bias assessment using a graph showing the reviewers’ judgments about each *risk of bias* domain in the *Results* section. We also showed reviewers’ judgments about each *risk of bias* domain for each included study using a figure in Multimedia Appendix 4 [10,20-24].

#### Data Synthesis

We synthesized the extracted data using narrative and statistical approaches. Specifically, meta-analysis was carried out when at least two studies assessed the same outcome of interest and reported sufficient data for the analysis (eg, mean difference, SD, and number of participants in each intervention group). When the abovementioned conditions were not met, we narratively synthesized findings of the included studies. We grouped and synthesized the findings according to the measured outcomes (ie, PA, glycemic control, and weight change).

We conducted a meta-analysis using Review Manager 5.4, which is a software developed by Cochrane. We used the mean difference to assess the effect of each trial and the overall effect when the outcome data were continuous, and the outcome measure of each outcome was identical in the meta-analyzed studies. However, we used the standardized mean difference when, among studies, the outcome was measured using different tools. We selected a random effects model in the analysis because of the clinical heterogeneity among the meta-analyzed studies in terms of intervention characteristics (eg, its directionality, purpose, and frequency) and population characteristics (eg, sample size and mean age).

We assessed the clinical heterogeneity of the meta-analyzed studies by inspecting the characteristics of their interventions, outcomes, participants, and comparators. Further, we evaluated the statistical heterogeneity of the meta-analyzed studies. To do so, we calculated a chi-square *P* value and *I*^2^ to evaluate the statistical significance of heterogeneity and degree of heterogeneity, respectively. We judged the meta-analyzed studies as heterogeneous when the chi-square *P* value was ≤.05 [25]. The degree of heterogeneity was considered unimportant, moderate, substantial, or considerable when *I*^2^ ranged from 0% to 40%, 30% to 60%, 50% to 90%, or 75% to 100%, respectively [25].

The overall quality of meta-analyzed evidence was examined using the Grading of Recommendations Assessment, Development, and Evaluation approach [26,27]. This approach assessed the quality of evidence based on five main criteria: risk of bias, inconsistency (ie, heterogeneity), indirectness, imprecision, and publication bias [26]. A total of 2 reviewers (MA and AA) independently assessed the overall quality of the meta-analyzed evidence, and any disagreements were resolved through discussion and consensus. Interrater agreement among the reviewers was very good (Cohen *κ*=0.81) [18].

## Results

### Search Results

**Figure 1:**
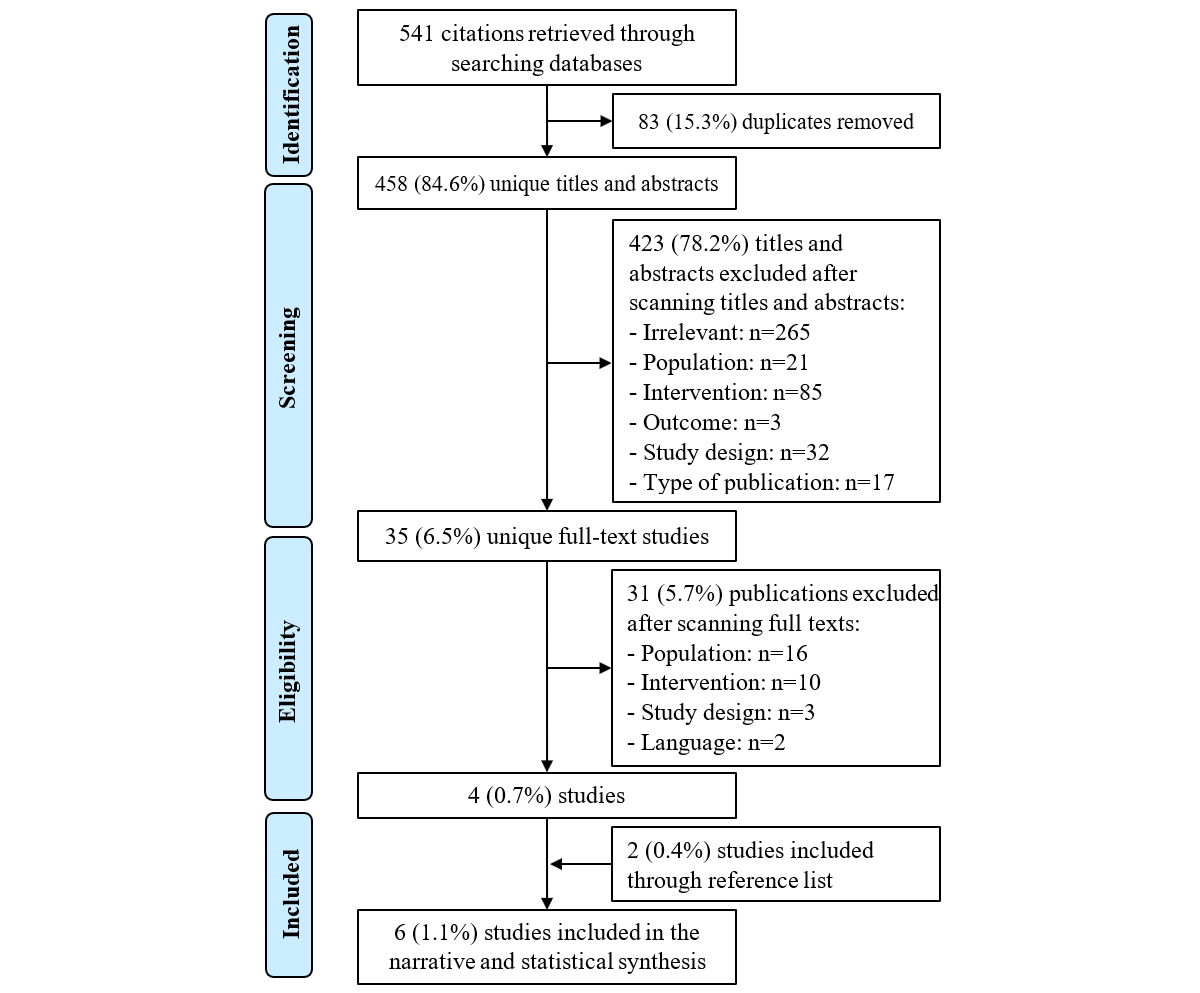
Flow chart of the study selection process.

We retrieved 541 citations by searching the 6 bibliographic databases (Figure 1). Of these 541 citations, 83 (15.3%) duplicates were identified and excluded. We screened the titles and abstracts of the remaining 84.6% (458/541) citations and excluded 78.2% (423/541) citations owing to reasons shown in Figure 1. By checking the full texts of the remaining 35 (6.5%) studies, 31 (5.7%) studies were not eligible for this review for several reasons (Figure 1). We identified 2 additional studies by backward reference list checking. Overall, we included 6 studies in this review [10,20-24]. At all steps, consensus was agreed between the 2 reviewers (MA and AA), and referral to a third reviewer was not required.

### Characteristics of Included Studies

As detailed in Table 1, all the included studies were RCTs. The included studies were conducted in 3 countries: the United States (n=3), Iran (n=2), and Indonesia (n=1); 4 of the studies were published in 2018. The sample size in the included studies ranged between 28 and 138, with an average of 81 (SD 40.03). The mean age of participants in the included studies varied from 44.6 to 65.5 years, with an average of 51.6 years (SD 6.7). The percentage of men in the included studies ranged from 23.3% to 57.9%, with an average of 42.2% (SD 12.1). All studies recruited patients with T2DM. The included studies recruited participants from health care (n=5) and community (n=1).

**Table 1:** Characteristics of studies and population.

Study	Year	Country	Study design	Sample size	Age (years), mean (SD)	Sex (male)	Health condition	Setting
Agboola et al [20]	2016	United States	RCT^a^	126	51.4 (11.5)	48.4%	T2DM^b^	Health centers
Arovah et al [21]	2018	Indonesia	RCT	43	65.5 (5.8)	37.2%	T2DM	Public hospital
Lari et al [10]	2018	Iran	RCT	73	47.6 (9.1)	53.4%	T2DM	Diabetes clinics
Lari et al [22]	2018	Iran	RCT	76	48.2 (8.8)	57.9%	T2DM	Diabetes clinics
Polgreen et al [23]	2018	United States	RCT	138	44.6 (15.9)	23.3%	T2DM	Community
Ramirez and Wu [24]	2017	United States	RCT	28	52 (9.0)	33%	T2DM	Ambulatory care clinic

^a^RCT: randomized controlled trial.

^b^T2DM: type 2 diabetes mellitus.

The interventions in the included studies were text messages only (n=1), text messages and educational CD about PA (n=1), and text messages and pedometers (n=4; Table 2). Text messages were unidirectional (n=1), bidirectional (n=4), and both (ie, most messages were unidirectional, and some messages were bidirectional; n=1). The purpose of the text messages in the included studies was to educate participants about PA (n=4), remind them to wear the pedometer, review goals, or self-monitor and record their steps (n=4), provide them with feedback about their previous day’s activity (n=3), motivate them to walk and exercise more (n=2), and set step goals (n=1). The frequency of text messages sent to participants ranged between 2 per week and 3 per day. The intervention was delivered for 12 weeks in 4 studies and 24 weeks in 2 studies. The intervention in 5 studies was theoretically informed. Specifically, the following theories or models were used to develop the intervention: Social Cognitive Theory (n=2), Health Promotion Models (n=2), and Transtheoretical Model and Grounded Theory (n=1).

**Table 2:** Characteristics of interventions.

Study	Intervention	Directionality	Purpose	Frequency	Period	Theory used
Agboola et al [20]	SMS and pedometers	1- and 2-way	Education, motivation, reminder, and feedback	2/day	24 weeks	Transtheoretical model and grounded theory
Arovah et al [21]	SMS and pedometers	2-way	Motivation and reminder	1-3/day	12 weeks	Social Cognitive Theory
Lari et al [10]	SMS	2-way	Education	Phase 1: 2-3/day; phase 2: 2/week	Phase 1: 2 weeks; Phase 2: 10 weeks	Health promotion models
Lari et al [22]	SMS + educational CD	1-way	Education	2/week	12 weeks	Health promotion models
Polgreen et al [23]	Intervention 1: SMS text messaging (reminder) + SMS text messaging (goal setting) + pedometer; intervention 2: SMS text messaging (reminder)+pedometer	2-way	Reminders, feedback, and setting goals	Intervention 1: 2/day; intervention 2: 1/day	24 weeks	N/A^a^
Ramirez and Wu [24]	Intervention 1: SMS text messaging + pedometer	2-way	Education reminders and feedback	≥4/week	12 weeks	Social Cognitive Theory

^a^N/A: not applicable.

The comparison group received pedometers in 4 of the studies or no intervention in 2 studies (Table 3). The pedometers were used by the participants for 12 weeks (n=2) or 24 weeks (n=2). The follow-up period ranged from 4 weeks to 24 weeks. The following outcomes of interest were assessed in the included studies: PA (n=6), glycemic control indicators (n=3), weight (n=1), and BMI (n=1). Step count was the most common outcome measure used in the included studies (n=4), followed by HbA_1c_ (n=2), weight scale (n=2), and metabolic equivalent of task questionnaire (n=2).

**Table 3:** Characteristics of comparators and outcomes.

Study	Comparator	Period (week)	Follow-up (week)	Outcome	Outcome measure
Agboola et al [20]	Pedometers	24	24	PA^a^, glycemic control, and weight	Step count, weight scale, and HbA_1c_^b^
Arovah et al [21]	Pedometers	12	12 and 24	PA and glycemic control	Step count, PAR^c^ questionnaire, HbA_1c_, fasting glucose, and 2-hour glucose
Lari et al [10]	No intervention	N/A^d^	4 and 12	PA	MET^e^ questionnaire
Lari et al [22]	No intervention	N/A	4 and 12	PA	MET questionnaire
Polgreen et al [23]	Pedometers	24	12 and 24	PA and BMI	Step count, weight scale, and stadiometer
Ramirez and Wu [24]	Pedometers	12	6 and 12	PA	Step count

^a^PA: physical activity.

^b^HbA_1c_: glycated hemoglobin.

^c^PAR: physical activity rating.

^d^N/A: not applicable.

^e^MET: metabolic equivalent of task.

### Risk of Bias Results

**Figure 2:**
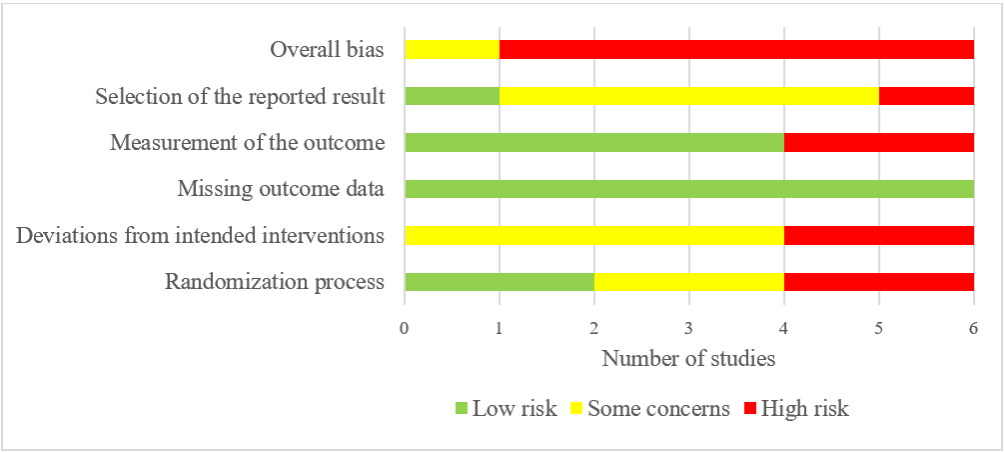
Review authors’ judgments about each risk of bias domain.

Although all studies used an appropriate random allocation sequence for the randomization process and had comparable groups, only 2 studies concealed the allocation sequence until participants were enrolled and assigned to interventions. Accordingly, only these 2 studies were rated as having a low risk of bias in the randomization process (Figure 2). In all studies, participants, their health care professionals, researchers, or individuals delivering the interventions were aware of the assigned intervention during the trial. The study also did not report any information about whether a deviation from the intended intervention occurred owing to the experimental context. Thus, none of the studies were rated as having a low risk of bias in deviations from the intended interventions (Figure 2).

Outcome data were not available for all participants in the included studies, and there was no evidence that the findings were not biased by missing outcome data. However, the reasons for missing outcome data were not related to the true value of the outcome in all studies. Thus, all studies were judged as having a low risk of bias in the domain of missing outcome data.

In 4 studies, the outcomes of interest were assessed using appropriate measures (eg, pedometer and HbA_1c_), which were comparable between the intervention groups. Therefore, these studies were rated as having a low risk of bias when measuring the outcome. However, the remaining 2 studies were judged as having a high risk of bias in this domain because they used subjective outcome measures that depended on participants’ recall, and participants and outcome assessors were not blinded in the 2 studies (Figure 2).

Only 1 study was judged as having a low risk of bias in the selection of the reported studies (Figure 2). This judgment is attributed to the fact that the remaining studies did not publish a prespecified analysis plan or reported outcome measurements and analyses different from those specified in the analysis plan. Given that 5 studies were judged as having a high risk of bias in at least one domain, they were rated as high risk in the domain of overall bias. The remaining study was judged to raise some concerns in the domain of overall bias, as it had some concerns in one of the domains. Reviewers’ judgments about each *risk of bias* domain for each included study are presented in Multimedia Appendix 4.

### Results of Studies

#### Effect on PA

All included studies assessed the effect of using text messages on PA among patients with T2DM. A total of 3 studies showed a statistically significant effect of text messages on PA [10,21,22,24]. To be more precise, Arovah et al [21] compared the effect of text messages plus pedometers to only pedometers on PA as measured by daily step count, self-reported walking (min/week), and self-reported moderate-to-vigorous-intensity PA (min/week). The study showed a statistically significant effect of 12-week text messages plus pedometers to only pedometers on daily steps (*P*<.001), self-reported walking (*P*=.001), and moderate-to-vigorous-intensity PA (*P*<.001) [21]. In 2 further studies, where data were analyzed from different arms of a single RCT in each *study*, Lari et al [10] compared the effect of text messages only and text messages plus educational CD [22] to no intervention on PA as measured by the metabolic equivalent of task questionnaire. Both studies found a statistically significant effect of text messages only (*P*<.001) [10] and text messages plus educational CDs (*P*<.001) [22] on PA compared with no intervention.

The 3 remaining studies did not find a statistically significant effect of text messages on PA [20,23,24]. Specifically, Agboola et al [20] compared the effect of text messages plus pedometers to pedometers only on PA, as measured by the monthly step count. Although the study found that step counts over 6 months were higher in the intervention group than in the control group, this difference was not statistically significant (*P*=.17) [20]. Another study assessed the effect of text messages plus pedometers and only pedometers on PA, as assessed by daily steps [24]. The study did not show any statistically significant difference (*P*=.78) in PA between the 2 groups [24]. In a previous study, Polgreen et al [23] compared the effect of 2 interventions to only pedometers on PA, as measured by daily step count. The first intervention was pedometers plus text message reminders to wear the pedometers (reminders and pedometers), whereas the second intervention was the same as the first intervention plus text messages asking participants to set a step goal (goal setting, reminders, and pedometers) [23]. The study found no statistically significant differences in PA among the 3 groups [23].

**Figure 3:**
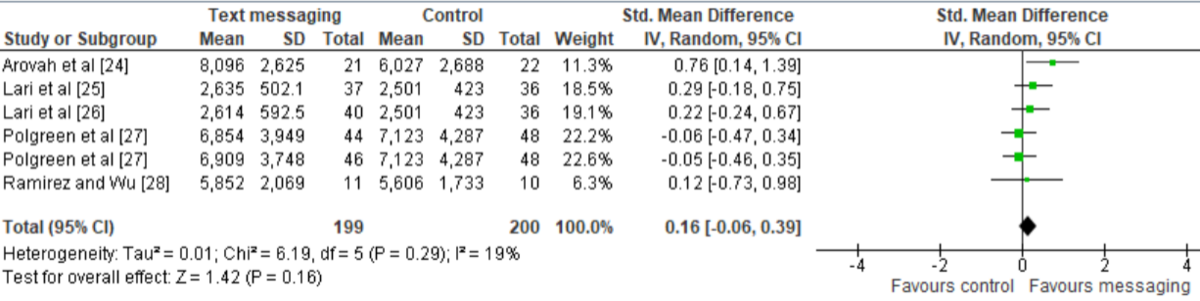
Forest plot of 6 studies assessing the effect of text messaging on physical activity.

A total of 5 studies were included in the statistical analysis (ie, meta-analysis), as they reported sufficient and appropriate data for the analysis [10,21-24]. The meta-analysis contained 6 comparisons as we included a comparison from each of the 4 studies [10,21,22,24] and 2 comparisons from the remaining study [23], which compared two types of text messages to no intervention. The meta-analysis showed no statistically significant difference in the PA (*P*=.16) between the text message group and the control group (standardized mean difference 0.16, 95% CI –0.06 to 0.39; Figure 3). The heterogeneity of the evidence was not a concern (*P*=.29; *I^2^*=19%). The quality of the evidence was very low because of the high risk of bias and impression (Multimedia Appendix 5).

#### Glycemic Control

**Figure 4:**
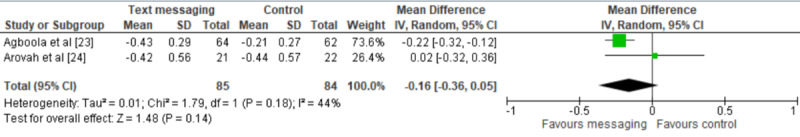
Forest plot of 2 studies assessing the effect of the text messaging on HbA_1c_.

A total of 2 studies examined the effect of text messages on glycemic control, as assessed by HbA_1c_ [20,21]. The results of both studies were meta-analyzed. The meta-analysis showed no statistically significant difference (*P*=.14) between the intervention and control groups, with no difference observed between text messages plus pedometers and only pedometers on HbA_1c_ (mean –0.16, 95% CI –0.36 to 0.05; Figure 4). There was moderate heterogeneity of the evidence (*I*^2^=44%), but the difference was not statistically significant (*P*=.18; Figure 4). The quality of evidence was low as it was downgraded by 1 level owing to a high risk of bias (Multimedia Appendix 5). It is worth mentioning that 1 of the 2 studies compared the effect of text messages plus pedometers with only pedometers on glycemic control as measured by fasting plasma glucose and 2-hour plasma glucose [21]. The study did not find a statistically significant difference between the groups in terms of fasting plasma glucose (*P*=.18) and 2-hour plasma glucose (*P*=.90) [21].

#### Anthropometric Measures

A total of 2 studies assessed anthropometric measures as outcomes (weight or BMI) [20,23]. The results of the 2 studies could not be statistically synthesized, as they assessed different outcomes. The first study showed no statistically significant difference between the intervention and control groups, with no effect of text messages plus pedometers on weight (*P*=.77) compared with pedometers alone [20]. In the second study, Polgreen et al [23] compared the effects of 2 interventions with only pedometers on BMI. The first intervention was pedometers plus text message reminders to wear the pedometers (reminders and pedometers), whereas the second intervention was the same as the first intervention plus text messages asking participants to set a step goal (goal setting, reminders, and pedometers) [23]. The study found no statistically significant differences in BMI among the 3 groups [23].

#### Other Outcomes

Secondary outcome measures reported in the examined studies included the following variables and parameters: reports of usability, satisfaction and adherence to the RCT as discussed in the study by Agboola et al [20], and quality of life or psychological outcomes (eg, self-efficacy, outcome expectations, self-regulation, and social support) as discussed in Arovah et al [21]. Lari et al [10,22] assessed the Health Promotion Model constructs (eg, perceived benefits, perceived barriers, perceived social support, and self-efficacy). Ramirez and Wu [24] also investigated the feasibility, perceived usefulness, and potential effectiveness.

## Discussion

### Principal Findings

This systematic review assessed the effectiveness of mobile text messaging as a method of promoting PA alone in people with T2DM. The meta-analysis of the results of 5 studies (6 comparisons) showed no statistically significant effect of mobile text messaging on PA in comparison with no intervention. The insignificant effect may be attributed to the fact that 3 studies showed a statistically significant effect of mobile text messaging on PA, whereas 2 studies did not find any significant effect of text messages on PA. There are several potential reasons for the significant increase in PA in 3 studies. First, the intervention in 1 study [21] was combined with pedometers, and some studies have found greater effects when using objective measures compared with subjective measures [28]. It is possible that participants in these studies were more active because of the knowledge that they were wearing the pedometer [29]. The remaining 2 RCTs [10,22] were rated as having a high risk of bias because they used self-recall questionnaires to measure PA. However, these measures can present limitations in capturing PA because of poor reliability and validity, participant recall bias, and differences in the interpretation of questions [30]. Our findings are consistent with previous reviews that assessed the effect of text messaging on PA in participants with different chronic conditions [31]. Some studies observed only small improvements in daily steps and self-reported PA; other studies did not observe any statistically significant changes in PA despite the use of different PA measurement strategies [31].

Our review found no statistically significant effect of mobile text messaging on glycemic control as assessed by HbA_1c_, fasting plasma glucose, and 2-hour plasma glucose. Our findings are consistent with those of previous studies that showed no significant difference in HbA_1c_ levels in people with T2DM following text messaging interventions [32]. This could be attributed to the duration effect, which had short interventions and follow-up durations (median 12 weeks); thus, outcomes such as HbA_1c_ are less likely to change over a short timescale (3 months). In other words, it might take longer for the intervention effects to become apparent [33].

The narrative synthesis in this review showed no statistically significant effect of mobile text messaging on either weight or BMI. We could not synthesize these measures in our meta-analysis because of the high heterogeneity in the included studies. Our findings are consistent with those of previous reviews, and a meta-analysis showed no statistically significant association between BMI and weight following mobile messaging interventions in people with T2DM [34]. However, it is important to be realistic about the period of intervention, and a longer period is required to determine the desired improvements in such clinical outcomes [35]. The aforementioned studies had short interventions (median 12 weeks); thus, outcomes such as weight and BMI are less likely to change on a short timescale [33].

### Strengths and Limitations

#### Strengths

Our study is the first review and meta-analysis that focused on the effectiveness of text messages targeting only PA among T2DM patients. This enabled us to ensure that the effect of text messaging on PA outcomes is attributed to PA-related message content and to no other content such as diet, lifestyle, and general diabetes education. Our study is considered a robust and high-quality review given that we followed well-recommended guidelines (ie, PRISMA) in developing, executing, and reporting it.

To run as sensitive a search as possible, we searched the most popular databases in the health and information technology fields using a very comprehensive list of search terms. The risk of publication bias is minimal in this review because we searched gray literature databases (ie, Web of Science and Google Scholar) and conducted backward and forward reference list checking. We did not restrict our search to specific countries of publication, year of publication, comparators, or settings; thus, this resulted in a more comprehensive review.

The risk of selection bias was minimal in the current review as 2 authors (MA and AA) independently selected the studies, extracted data, and assessed the risk of bias and quality of evidence, and they had a very good interrater agreement in all processes. When possible, we meta-analyzed the results of the included studies, and this improved the power of studies and the estimates of the likely size of the effect of text messaging on different outcomes.

#### Limitations

The intervention of interest in this review was restricted to PA-related text messaging, so we did not examine the impact of other digital interventions, such as mobile apps, wearables, or other eHealth tools. We also focused on patients with T2DM, rather than patients with other types of diabetes. Accordingly, our results may not be generalizable to other eHealth interventions or patients with type 1 diabetes mellitus or gestational diabetes mellitus. In this review, we included only RCTs published in the English language; thus, it is possible that we missed results from some non-English RCTs. We applied these restrictions owing to the high internal validity of RCTs over other study designs [36] and lack of resources to translate non-English studies. The included studies were conducted in only 3 countries (the United States, Iran, and Indonesia); therefore, the generalizability of our findings to other countries may be limited. The findings were based on a small number of studies that met the review criteria. Although 6 studies were included in this review, 2 (33%) of the studies were from a single RCT where 2 separate analyses were undertaken with data taken from different arms. Only 2 studies were included in each of the 2 meta-analyses conducted in this review. This is attributed to the lack of reported data that were appropriate for the analysis and incomparable outcome measures and comparators between studies. As such, it is not possible to draw firm conclusions about effectiveness.

### Implications for Research

The current review found relatively few studies assessing the effectiveness of text messages in promoting PA in T2DM; thus, RCTs with larger sample sizes are needed. Future studies should seek to include objective outcome measures (eg, PA, glycemic control, and anthropometric measures), be consistent in terms of selected outcome measures, and measure outcomes after longer follow-up periods to be able to compare study findings and make firm conclusions about intervention effectiveness. More research is needed to determine the type of text message content, frequency of messaging, and duration of intervention that is most likely to result in positive outcomes. Additional research needs to include an estimation of the cost-effectiveness of text messages and an examination of their long-term impact.

### Conclusions

We could not draw a definitive conclusion regarding the effectiveness of text messaging on PA, glycemic control, weight, or BMI among patients with T2MD, given the low number of included studies and their high risk of bias. Thus, the findings of this study suggest that texting messaging should not substitute but rather supplement clinical support. In addition, there is a pressing need for further RCTs with large sample sizes, low risk of bias, and more consistency regarding intervention duration, outcome measures, follow-up period, and comparator.

## Appendix

Multimedia Appendix 1 PRISMA (Preferred Reporting Item for Systematic Reviews and Meta-Analyses) checklist.

Multimedia Appendix 2 Search query used for searching MEDLINE.

Multimedia Appendix 3 Data extraction form.

Multimedia Appendix 4 Reviewers’ judgements about each “risk of bias” domain for each included randomized controlled trial.

Multimedia Appendix 5 Grading of Recommendations Assessment, Development, and Evaluation profile.

## Abbreviations

HbA_1c_: glycated hemoglobin
PA: physical activity
PRISMA: Preferred Reporting Item for Systematic Reviews and Meta-Analyses
RCT: randomized controlled trial
T2DM: type 2 diabetes mellitus
