# Supplementary material for: The Effectiveness of Mobile Phone Messaging–Based Interventions to Promote Physical Activity in Type 2 Diabetes Mellitus: Systematic Review and Meta-analysis
Source: J Med Internet Res. 2022 Mar 8;24(3):e29663. doi: 10.2196/29663 (PMC8941442; doi:10.2196/29663)
Supplement: Multimedia Appendix 2 [file jmir_v24i3e29663_app2.docx]

**Appendix 2 Search query used for searching MEDLINE**

Database(s): **Ovid MEDLINE(R) and Epub Ahead of Print, In-Process & Other Non-Indexed Citations and Daily**1946 to June 19, 2020
Search Strategy:

| **#** | **Searches** | **Results** |
| --- | --- | --- |
| 1 | exp Diabetes Mellitus, Type 2/ | 132212 |
| 2 | Type 2 diabetes.tw. | 124203 |
| 3 | Diabetes type 2.tw. | 1212 |
| 4 | T2DM.tw. | 20337 |
| 5 | Type II diabetes.tw. | 8291 |
| 6 | Non-insulin-dependent.tw. | 10868 |
| 7 | Non-insulin-dependent diabetes.tw. | 8617 |
| 8 | NIDDM.tw. | 6928 |
| 9 | Ketoacidosis-resistant diabetes.tw. | 1 |
| 10 | Adult-onset diabetes.tw. | 443 |
| 11 | Maturity-onset diabetes.tw. | 1786 |
| 12 | Mature-onset diabetes.tw. | 24 |
| 13 | exp Text Messaging/ | 2838 |
| 14 | Text messag*.tw. | 4063 |
| 15 | Short messag*.tw. | 1283 |
| 16 | SMS messag*.tw. | 199 |
| 17 | Short message service*.tw. | 1041 |
| 18 | exp Exercise/ | 193923 |
| 19 | Physical exercis*.tw. | 16361 |
| 20 | Physical training.tw. | 5701 |
| 21 | Physical activit*.tw. | 110312 |
| 22 | Sport activit*.tw. | 1647 |
| 23 | Physical fitness.tw. | 9248 |
| 24 | Physical performance.tw. | 9124 |
| 25 | Physical effort.tw. | 1304 |
| 26 | Physical inactivity.tw. | 7717 |
| 27 | Physical work*.tw. | 3958 |
| 28 | Glycated Hemoglobin A/ | 34786 |
| 29 | HbA1C.tw. | 33430 |
| 30 | fasting plasma glucose.tw. | 12486 |
| 31 | fasting glucose.tw. | 16936 |
| 32 | glycaemic control.tw. | 8541 |
| 33 | Body Mass Index/ | 125998 |
| 34 | "Weights and Measures"/ | 2558 |
| 35 | Step count*.tw. | 1840 |
| 36 | Randomized Controlled Trials as Topic/ | 133923 |
| 37 | randomized controlled trial*.tw. | 134443 |
| 38 | randomised controlled trial*.tw. | 43092 |
| 39 | randomized control trial*.tw. | 6779 |
| 40 | randomised control trial*.tw. | 1722 |
| 41 | experiment*.tw. | 2040984 |
| 42 | 1 or 2 or 3 or 4 or 5 or 6 or 7 or 8 or 9 or 10 or 11 or 12 | 188193 |
| 43 | 13 or 14 or 15 or 16 or 17 | 5758 |
| 44 | 18 or 19 or 20 or 21 or 22 or 23 or 24 or 25 or 26 or 27 or 28 or 29 or 30 or 31 or 32 or 33 or 34 or 35 | 470104 |
| 45 | 36 or 37 or 38 or 39 or 40 or 41 | 2305661 |
| 46 | 42 and 43 and 44 and 45 | 64 |
