# Supplementary material for: The Effectiveness of Mobile Phone Messaging–Based Interventions to Promote Physical Activity in Type 2 Diabetes Mellitus: Systematic Review and Meta-analysis
Source: J Med Internet Res. 2022 Mar 8;24(3):e29663. doi: 10.2196/29663 (PMC8941442; doi:10.2196/29663)
Supplement: Multimedia Appendix 3 [file jmir_v24i3e29663_app3.docx]

| **Concept** | **Definition** |
| --- | --- |
| **Study Characteristics** |  |
| Author | The first author of the study. |
| Year of Publication | The year in which the study was published. |
| Country of publication | The country where the study was published. |
| Study design | The research method that the study used to collect the data (e.g. RCTs) |
| Study aim | What the study aimed to find out. |
| **Population characteristics** |  |
| Number of participants | Number of people who participated in the study. |
| Mean age | The average age of participants. |
| Gender (male) | Percentage of males in the sample. |
| Health condition | Health status of participants. |
| Recruitment Setting | Place where participants were recruited (i.e. clinical, educational, community settings). |
| **Intervention characteristics** |  |
| Intervention | What is the intervention of interest in the study |
| Directionality | Modality of text message in terms of direction of messages:   1. Unidirectional (1-way): participants can receive messages but cannot send or reply to them. 2. Bidirectional (2-way): participants can receive and send messages |
| Purpose | What is the aim of the intervention (providing education, motivation, reminders, feedback, etc.) |
| Frequency | How frequent was the intervention delivered to participants (e.g., 3 times/day, 2 times/ week) |
| Period | How long was the intervention provided (e.g. 12 weeks, 24 weeks) |
| **Comparator** **Characteristics** |  |
| Comparator | What is the comparator (e.g. usual care, pedometers, not intervention)? |
| Frequency | How frequent was the comparator delivered to participants (e.g., 3 times/day, 2 times/ week) |
| Period | How long was the comparator provided (e.g. 12 weeks, 24 weeks) |
| **Outcome characteristics** |  |
| Measured outcome | What was the outcome that the study measured? |
| Outcome measure | What is the tool used for measuring the outcome? |
| Follow-up period | When was the outcome measured? |
| **Findings** |  |
| Results of the study | Any reported statistics related to the outcome of interest, e.g. numbers, means, mean differences, standard deviations, confidence intervals, and *P*-values. |

**Appendix 3: Data extraction form**
