# Supplementary material for: The Effectiveness of Mobile Phone Messaging–Based Interventions to Promote Physical Activity in Type 2 Diabetes Mellitus: Systematic Review and Meta-analysis
Source: J Med Internet Res. 2022 Mar 8;24(3):e29663. doi: 10.2196/29663 (PMC8941442; doi:10.2196/29663)
Supplement: Multimedia Appendix 4 [file jmir_v24i3e29663_app4.docx]

**Appendix 4: Reviewers’ judgements about each “risk of bias” domain for each included study**


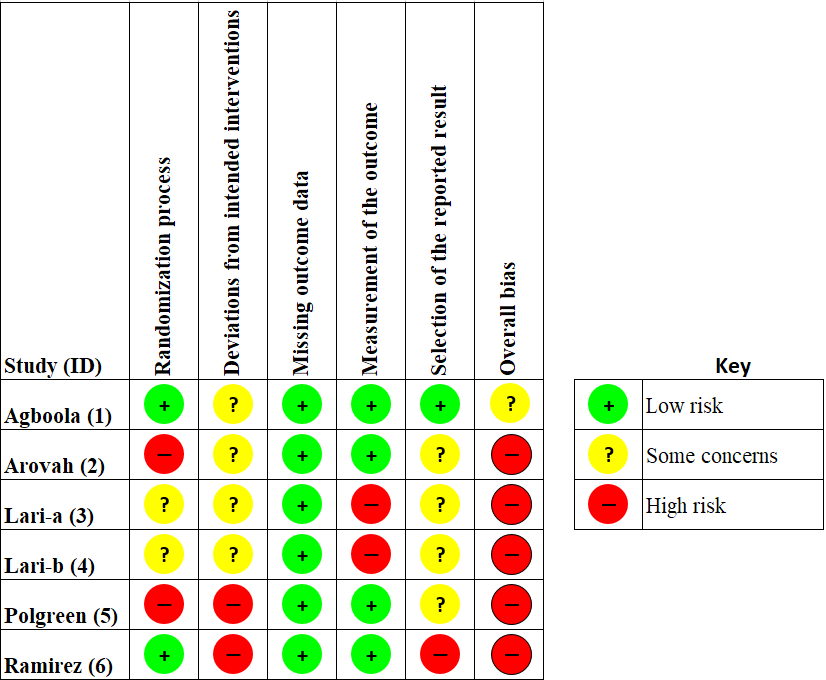


**References**

1. Agboola S, Jethwani K, Lopez L, Searl M, O'Keefe S, Kvedar J. Text to Move: A Randomized Controlled Trial of a Text-Messaging Program to Improve Physical Activity Behaviors in Patients With Type 2 Diabetes Mellitus. J Med Internet Res. 2016 Nov 18;18(11):e307. PMID: 27864165. doi: 10.2196/jmir.6439.

2. Arovah NI, Kushartanti BMW, Washington TL, Heesch KC. Walking with Diabetes (WW-DIAB) programme a walking programme for Indonesian type 2 diabetes mellitus patients: A pilot randomised controlled trial. SAGE Open Medicine. 2018;6. doi: 10.1177/2050312118814391.

3. Lari H, Noroozi A, Tahmasebi R. Impact of Short Message Service (SMS) Education Based on a Health Promotion Model on the Physical Activity of Patients with Type II Diabetes. Malays J Med Sci. 2018 May;25(3):67-77. PMID: 30899188. doi: 10.21315/mjms2018.25.3.7.

4. Lari H, Tahmasebi R, Noroozi A. Effect of electronic education based on health promotion model on physical activity in diabetic patients. Diabetes Metab Syndr. 2018 Jan - Mar;12(1):45-50. PMID: 28869152. doi: 10.1016/j.dsx.2017.08.013.

5. Polgreen LA, Anthony C, Carr L, Simmering JE, Evans NJ, Foster ED, et al. The effect of automated text messaging and goal setting on pedometer adherence and physical activity in patients with diabetes: A randomized controlled trial. PLoS One. 2018;13(5):e0195797. PMID: 29718931. doi: 10.1371/journal.pone.0195797.

6. Ramirez M, Wu S. Phone Messaging to Prompt Physical Activity and Social Support Among Low-Income Latino Patients With Type 2 Diabetes: A Randomized Pilot Study. JMIR Diabetes. 2017 Jun 6;2(1):e8. PMID: 30291094. doi: 10.2196/diabetes.7063.
