# Supplementary material for: The Effectiveness of Mobile Phone Messaging–Based Interventions to Promote Physical Activity in Type 2 Diabetes Mellitus: Systematic Review and Meta-analysis
Source: J Med Internet Res. 2022 Mar 8;24(3):e29663. doi: 10.2196/29663 (PMC8941442; doi:10.2196/29663)
Supplement: Multimedia Appendix 5 [file jmir_v24i3e29663_app5.docx]

**Appendix 5: GRADE Profile for comparison of text messaging to control for Type 2 Diabetes**

| Certainty assessment | | | | | | № of patients | | Effect | | **Certainty** |
| --- | --- | --- | --- | --- | --- | --- | --- | --- | --- | --- |
| **Participants (studies)** | **Risk of bias** | **Inconsistency** | **Indirectness** | **Imprecision** | **Other considerations** | **Text messaging** | **controls** | **Relative (95% CI)** | **Absolute (95% CI)** |  |
| **Physical activity** | | | | | | | | | | |
| 399 (5 RCTs) | very serious ^a^ | not serious | not serious | serious ^b,c^ | none | 199 | 200 | - | SMD **0.16 higher** (0.06 lower to 0.39 higher) | ⨁◯◯◯ VERY LOW |
| **HbA1C** | | | | | | | | | | |
| 169  (2 RCTs) | very serious ^a^ | not serious ^b^ | not serious | not serious | none | 85 | 84 | - | MD **0.16 lower**  (0.36 lower to 0.05 higher) | ⨁⨁◯◯ LOW |

**CI:** Confidence interval; **MD:** Mean difference

#### Explanations

a. Evidence was downgraded by 2 levels because the overall risk of bias was rated as high in all studies due to issues mainly in the randomization process, selection of the reported results, and deviations from intended interventions.

b. Evidence was downgraded by 1 level because 95% CI crosses one of MID boundaries for this outcome.

c. MID for this outcome, calculated as ± 0.5 times the standardized mean difference (SMD), is ± 0.08.
